# Do interventions principally targeting excessive alcohol use in young people improve depression symptoms?: a systematic review and meta-analysis

**DOI:** 10.1186/s12888-022-04006-x

**Published:** 2022-06-21

**Authors:** Kim Fredman Stein, Jennifer L. Allen, Ross Robinson, Cassandra Smith, Katherine Sawyer, Gemma Taylor

**Affiliations:** 1 Department of Psychology, University of Bath, 10 West, Bath, BA2 7AY UK; 2 Addiction and Mental Health Group (AIM), Department of Psychology, University of Bath, Bath, UK

**Keywords:** Alcohol, Depression, Young people, Systematic review, Meta-analysis

## Abstract

**Background:**

Excessive alcohol use is common in young people and is associated with a range of adverse consequences including an increased risk of depression. Alcohol interventions are known to be effective in young people, however it is not known if these interventions can also improve depression.

**Objective:**

To investigate whether psychosocial interventions principally targeting excessive alcohol use in young people reduce depression symptoms compared to controls.

**Design:**

We conducted a systematic review and meta-analysis of controlled intervention trials, that measured depression symptoms at follow-up. We used a generic inverse variance random effect meta-analysis to pool the standardised mean difference in change in depression symptoms from baseline to follow-up between intervention and control arms. We used I^2^ to measure heterogeneity, the Cochrane tool for randomised trials to assess risk of bias, and Egger’s tests to assess small study bias.

**Data sources:**

APA PsycNET, PubMed, Cochrane Central Register of Controlled Trials, Web of Science, Embase (including MEDLINE), and clinicaltrials.gov were searched for relevant studies published from inception to December 2020. Reference lists of studies were also searched, and authors contacted where articles presented insufficient data.

**Study eligibility criteria:**

Intervention studies that primarily targeted existing excessive alcohol use in young people (aged 10 to 24) and assessed depression outcomes at baseline with a minimum of four-week follow-up.

**Results:**

Five studies were included in the meta-analysis. Interventions targeting excessive alcohol use were associated with a reduction in depression symptoms from baseline to follow-up when compared to control, standardised mean difference = − 0.26, and 95% confidence interval [− 0.41, − 0.12], *p* < .001.

**Conclusions:**

This study found evidence that interventions primarily targeting excessive alcohol use can reduce depression symptoms in young people. However, this finding should be taken with caution given concerns about risk of bias in all studies. More research is needed to examine whether these findings generalise beyond populations of undergraduate students primarily living in high income countries.

**Trial registration:**

PROSPERO registration number: CRD42020177260.

**Supplementary Information:**

The online version contains supplementary material available at 10.1186/s12888-022-04006-x.

## Introduction

Excessive alcohol use is common among young people (defined as age 10 to 24; [35]) and is associated with a range of adverse outcomes, including disability and premature death [13]. The term ‘excessive alcohol use’ (defined as drinking over the recommended limit) encompasses all forms of alcohol misuse or excessive drinking, including terms in the literature such as alcohol abuse, alcohol-related harm, hazardous drinking, binge drinking, and alcohol use disorder [5]. According to the US National Survey on Drug Use and Health in 2017, almost 14 million 12 to 25-year-olds engaged in excessive alcohol use in the past month. This constitutes 5.3% of 12- to 17-year-olds, and 36.9% of 18 – 25-year-olds [30]. The UK Office of National Statistics (ONS, 2017) found that approximately 10% of secondary school pupils surveyed (aged 11-15) used alcohol excessively in the last week, and 37.3% of young people aged 16-24 reported binge drinking (exceeding 6-8 units on 1 day), which was significantly higher than any other age group surveyed.

Excessive alcohol use during adolescence and young adulthood has been found to predict later alcohol use disorders (e.g., [9]). Additionally, excessive alcohol use has consistently been associated with mood disturbance and depressive symptoms among young people [26, 28]. At the more severe end of the continuum, there is high comorbidity between alcohol use disorder and major depressive disorder in young people [26]. Evidence suggests that this comorbidity is particularly debilitating; young people with comorbid major depressive disorder and alcohol use disorder are more likely to have lower global functioning, life satisfaction and are more likely to attempt suicide than those with either major depressive disorder or alcohol use disorder alone [3]. A review investigating the association between alcohol use disorders and major depression found evidence that excessive alcohol use is associated with an increased risk of depression, but that this relationship is likely bidirectional [2]. The authors attributed the link between excessive alcohol use and an increased risk of later depression to the metabolic, neurophysiological and circadian rhythm changes which result from alcohol use. The negative impact of excessive alcohol use on young people’s quality of life is another mechanism that may explain increased depression symptoms [22]. The findings of this review are consistent with evidence indicating that excessive alcohol use can precede an increase in depression symptoms. For example, results from UK surveys indicate that individuals who abstained from alcohol were less likely to become depressed in the following 18-months (odds ratio [OR] = 0.36, 95% CI [0.17–0.77]; [14]). Moreover, problematic alcohol use has been found to be associated with a greater severity of depression symptoms over time, as well as an increased risk of suicide [31]. Regardless of the direction of causality, it is important to understand the impact of interventions primarily targeting alcohol use on depression, as well as alcohol outcomes.

Several randomised controlled trials targeting excessive alcohol use in young people have included depression as an outcome. However, to-date, no systematic review has investigated the impact of these interventions on depression outcomes. For example, Calabria et al. [4] conducted a systematic review to evaluate interventions for young people with alcohol use problems, however, they did not synthesise depression outcomes. A more recent systematic review by Hobden et al. [18] examined the efficacy of integrated treatment models for depression and alcohol misuse versus single focused treatment models. They found little evidence that integrated treatments are superior to single focused treatments in relation to either depression or alcohol misuse. However, they did not investigate the efficacy of single focused alcohol use interventions versus controls.

Given the strong link between excessive alcohol use and depression (e.g., [28]), and evidence in support of less resource intensive treatments for excessive alcohol use in young people [32], it would be useful to know whether single focused psychosocial interventions targeting excessive alcohol use also improve depression outcomes compared to controls. From a cost and scalability point of view, this is important because single focused interventions generally involve briefer interventions which can be effectively delivered by non-specialist practitioners, while the integrated interventions generally involve more complex psychological interventions for depression such as Cognitive Behavioural Therapy.

Understanding whether psychosocial interventions targeting excessive alcohol use improve depression in young people specifically (ages 10-24; [35]) is important for several reasons. This period encompasses key stages for development in important domains (for example, for the development of executive functioning and emotional regulation; [29]), and excessive alcohol use during ongoing neurodevelopment has known psychological, neurological and physical consequences [17]. For example, higher consumption of alcohol use in young people is associated with poorer executive functioning compared to controls and individuals who engage in excessive alcohol use later in life [33]. It is also a time in which peer relationships are of increased importance, and this can elevate the risk of excessive alcohol use due to peer pressure [1]. Moreover, excessive alcohol use before the age of 15 increases the risk of both major depression and chronic substance use disorder later in life [6], while comorbid alcohol misuse and depression in young people is associated with lower psychosocial functioning at age 30 [3].

Thus, it is critical to understand whether psychosocial interventions (i.e., interventions targeting psychological and/or social factors, such as psychological therapies, counselling, behavioural interventions or equivalent) targeting excessive alcohol use in young people lead to reductions in depression symptoms for several reasons: (1) theoretically, in order to elucidate the nature of the link between excessive alcohol use and depression, (2) clinically, in identifying scalable treatments, and (3) in shaping policy, to minimise the negative effects at these key developmental stages. Therefore, this systematic review aimed to examine whether psychosocial interventions principally targeting excessive alcohol use in young people reduce depression symptoms compared to controls.

## Methods

The study protocol was published on PROSPERO (CRD42020177260), and PRISMA reporting guidelines were followed [19].

### Eligibility criteria

The following eligibility criteria were used:

- Population: Young people aged 10 to 24 years [35] with existing excessive alcohol use, as defined by drinking over recommended limits (more than four drinks on 1 day or more than 14 drinks per week for men, more than three drinks on 1 day and more than seven drinks per week for women, or drinking underage; [23])
- Intervention: Psychological or psychosocial interventions primarily targeting existing excessive alcohol use i.e., studies that include the primary treatment target as alcohol use and/or have alcohol use as a primary outcome measure.
- Control: Any (including active and non-active controls)
- Outcome: Depression symptoms measured continuously (mean change in depression symptoms from baseline to follow-up)
- Study design: Psychological or psychosocial intervention trials (randomised controlled trials, non-randomised trials and non-controlled trials). Where studies presented multiple follow-ups, the longest follow-up data available was used, as recommended by the Cochrane Collaboration [15].

### Information sources and searches

APA PsycNET, PubMed, Cochrane Central Register of Controlled Trials, Web of Science, Embase (including MEDLINE), and clinicaltrials.gov were searched for studies published from inception to December 2020. The last date searched was 18th December 2020. Both published and unpublished studies and trials (such as conference proceedings) were included in the search to reduce the impact of publication bias, for example by using clinicaltrials.gov. Reference lists of studies included were also searched for appropriate articles. A combination of search terms relating to alcohol, psychological or psychosocial interventions, young people, depression and study design were used (see Additional file 1: Appendix 1A). Where full texts were not available (e.g., for conference abstracts), contact was made with study authors to request them. Duplicates were automatically removed by Covidence software.

### Study selection

In the first stage, all titles and abstracts were screened for eligibility, and 30% of these were screened again independently by a second researcher to reduce the possibility of error. In the second stage, those studies included from the title and abstract screening stage were read in full by both the primary researcher and again independently by a second researcher. In order to maximise sensitivity, studies were included in the full-text screening if there was insufficient information in the abstract to assess a study’s eligibility. The primary and secondary researchers met regularly to discuss disagreements and queries relating to the inclusion or exclusion of each paper.

### Data collection process

The data extraction form was piloted before use, and changes were made. Data extraction of the effect estimate and its variance was done by both the primary author, and independently by a second researcher, to control for error. Where data on change in depression score from baseline to follow-up for intervention and control groups, and its variance, were not available, authors were contacted to request this data. Studies were excluded from the meta-analysis, but included narratively, if this data were not provided.

### Data items

- Participants: Measure of depression symptoms, criteria for minimum depression symptoms, criteria for minimum consumption of alcohol, participant age (mean and standard deviation), gender, level of education and ethnicity (where reported) were recorded.
- Intervention: Type of intervention and length of intervention were extracted.
- Control: Type of control and length of control were extracted.
- Outcome: Data on change in depression symptoms from baseline to follow-up between intervention and control groups were extracted (see statistical methods for more information).
- Other items: Additional data required by the Cochrane risk of bias tool for randomised trials [16] were also extracted to assess risk of bias.

### Statistical methods

The summary measure used was the standardised mean difference (SMD) in change in depression scores between intervention and control groups from baseline to follow-up. The SMD was used as depression scales varied between studies, in line with recommendations for meta-analysis from the Cochrane Collaboration [15]. The 95% confidence interval was also extracted, and the standard error (SE) was calculated. Where the SMD was unavailable, effect estimate extraction was prioritised in the following order based on availability of data: (1) Mean change in depression from baseline to follow-up and its variance for intervention and control groups; (2) Baseline and follow-up mean depression scores and variance for intervention and control groups. These values were then used to calculate the SMD and standard error using standard formulas stipulated by the Cochrane Collaboration [15]. For example, if a study presented means at baseline and at follow-up for each trial arm and their variance, we first calculated the mean change, and second calculated the difference in change and its variance. If a study presented the mean change and its variance for each arm, we then calculated the difference in change and its variance. All formula used are available in the Cochrane Handbook [15].

### Meta-analysis method

A generic inverse variance random effects model was used to pool the SMD in change in depression symptoms from baseline to follow-up between intervention and control arms. This was selected to incorporate heterogeneity between and within studies, including heterogeneity of follow-up period. I^2^ was used to quantify statistical heterogeneity. RevMan 5.4 was used to conduct the meta-analysis, as well as the subgroup and sensitivity analysis. Forest plots were generated using RevMan 5.4.

### Risk of bias assessment

The Cochrane risk-of-bias tool for randomised trials [16] was used to assess risk of bias within studies at study level. Specifically, the tool was used to assess the effect of assignment to intervention. The tool’s risk categories are: risk of bias arising from the randomisation process, risk of bias due to deviations from the intended intervention, risk of bias due to missing outcome data, risk of bias due to measurement of the outcome, and risk of bias in selection of the reported result. Two researchers separately rated each study and met to discuss any disagreements. Where disagreements could not be resolved a third researcher was consulted until an agreement was met.

### Small study bias

Eggers tests were conducted to investigate small study bias.

### Protocol deviations

Although a three-month follow-up was specified in the study protocol, on review this was reduced to 1 month given that three reasonably robust studies which met all other inclusion criteria only used a one-month follow-up. It was decided to include these studies but to conduct a sensitivity analysis to investigate whether the pooled effect estimate was affected by including studies with a shorter follow-up.

## Results

### Search results

**Fig. 1:**
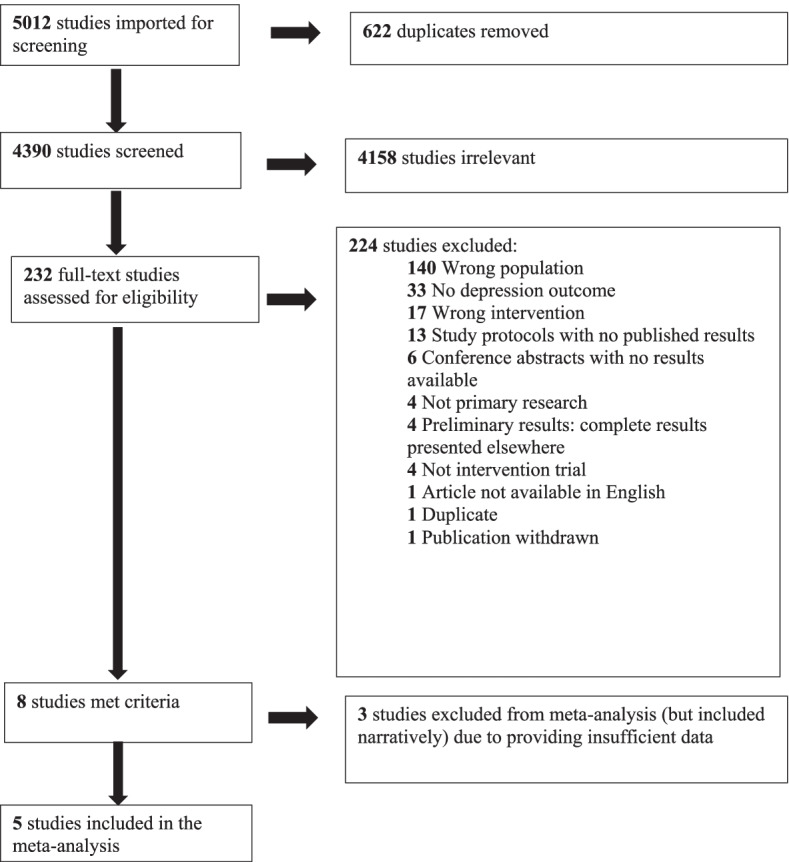
Flow Diagram

The database and reference list searches yielded 4390 studies, after 622 duplicates had been removed. Following title and abstract screening, 232 full text articles were assessed for eligibility, and of these, 224 were excluded before data extraction (see Fig. 1 for a diagram with the reasons for exclusion at each stage). Three studies were excluded from the meta-analysis at the data extraction stage as they provided insufficient data, but these studies supplied sufficient details to be included in a narrative synthesis. Five studies were included in the meta-analysis.

### Study characteristics

#### Studies included in the meta-analysis

All five studies included in the meta-analysis were randomised controlled trials (RCTs) of students in university settings. Four RCTs were conducted in the USA, and one was conducted in South Africa. The average participant age was 20.2 years and sample sizes ranged from 82 to 393 participants. Three studies used a one-month follow-up period, one study used a 12-month follow-up period, and one study used a 16-month follow-up period. Interventions included a brief counselling session on alcohol risk reduction, brief motivational interventions plus substance free activity scheduling interventions, and a web-based intervention using psychoeducation and personalised feedback. Controls ranged from feedback on alcohol screening and a leaflet on responsible drinking, assessment only control, and brief motivational intervention plus relaxation training. In one study, both the intervention and control groups were given CBT for depression, while the intervention group was additionally given brief motivational interviewing. Depression symptoms were measured using a range of self-report measures (see Additional file 1: Appendix 1B for further details).

#### Studies included in the narrative synthesis

Of the studies included in the narrative synthesis, two were RCTs and one was a pilot randomised trial. Sample size ranged from 69 to 986 participants, and all studies measured depression through self-report measures. All were conducted in North America; one study enrolled college students, one enrolled patients aged 14-20 in an emergency department, and one enrolled young people who self-identified as American Indian/Alaska Natives. The mean age of participants in the two studies was 19.8 years (the third study did not report a mean age). Interventions all included aspects of motivational interviewing, and controls were either psychoeducation or enhanced usual care. Length of follow-up ranged from 12-months to 2 years (see Additional file 1: Appendix 1C for further details).

### Risk of bias

Application of the Cochrane Risk of Bias tool indicated that there were “some concerns” about all five studies included in the meta-analysis (see Additional file 1: Appendix 1D). Thus, there is plausible risk of bias which raises some doubt about the results both within and across studies. Of the studies included narratively, there were “some concerns” about two studies, and one was judged to be at a high risk of bias (Additional file 1: Appendix 1D). For the study at a high risk of bias [12], this indicates plausible bias that significantly weakens confidence in the results of this study [15].

One study included in the narrative synthesis [10] only provided data for their significant findings (for example, for alcohol use), but did not provide data for the non-significant findings, including for depression (an example of publication bias), and so could not be included in the meta-analysis. All studies were published in peer-reviewed journals, thus it is possible that there may have been other unpublished studies which were not included. For example, several conference abstracts were found through the systematic search, for which the full results were not available, despite attempts to contact authors.

### Publication and outcome reporting bias

An Egger test indicated no small study bias (*p* = 0.169).

### Results of the meta-analysis

**Fig. 2:**
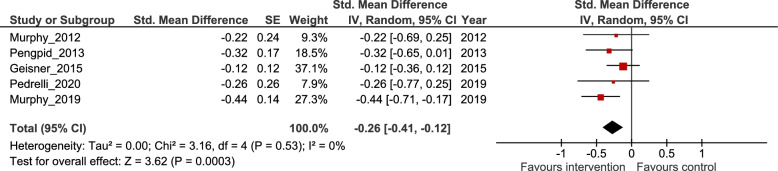
Forest Plot Showing Mean Change in Depression Score Between Intervention and Control Groups from Baseline to Follow-Up. Note. SE = standard error, CI = confidence interval

Figure 2 shows [11, 20, 21, 25, 27] the difference in mean change in depression scores from baseline to follow-up between interventions principally targeting alcohol use as compared to control. Compared with controls, interventions targeting excessive alcohol use reduced depression symptoms from baseline to final follow-up, SMD = − 0.26, 95% CI [− 0.41, − 0.12], *p* < .001; I^2^ = 0%. The confidence interval does not include the null, and I^2^ indicates that there was no evidence of statistical heterogeneity.

### Sensitivity analysis of length to follow-up

After removing the two studies with less than a 3-month follow-up, the effect of interventions targeting excessive alcohol use on depression symptoms remained when compared with controls, SMD = − 0.36, 95% CI [− 0.56, − 0.17], *p* < .001, I^2^ = 0%.

### Results of narrative synthesis

Three studies were excluded because they presented insufficient data to extract an effect estimate or its variance, despite attempts to contact authors. One study [24] found that depression improved significantly more in the intervention groups at three-month follow-up (both in the therapist-delivered and computer-based conditions) and at 6 months (in the computer-based condition only), but there was no difference at the 12-month follow-up in either of the two intervention conditions compared to control. A second study [10] found no significant differences in depression scores between the intervention and control groups at a 12-month follow-up but did not report the results of this analysis. A third study [12], found equivocal results for the intervention and control groups when men and women were examined together, but women in the motivational interviewing group reported significantly less depression than women in the control group at two-year follow-up.

## Discussion

This study found evidence that psychosocial interventions principally targeting excessive alcohol use in young people can improve depression symptoms. This indicates that alcohol use interventions can be helpful for reducing symptoms of depression as well as alcohol use in populations of young people. However, this finding should be interpreted with caution as the pooled effect estimate was relatively small (SMD = 0.26) and none of the studies included were judged to be at low risk of bias.

### Comparison to other studies and possible mechanisms

The effect estimate found in the current study is lower than effect estimates found in a meta-analysis of psychotherapies targeting depression in adolescents (*g* = 0.55, 95% CI, [0.34-0.75]), and in young adults (*g* = 0.98, 95% CI, [0.79-1.16]); [7]). However, given the suboptimal quality of and high heterogeneity of the studies included, the effect estimates from Cuijpers et al. [7] meta-analysis should be taken with caution. The effect estimate found in the current study is also lower than effect estimates found for fluoxetine in young people (SMD = − 0.51, 95% CI [− 0.99, − 0.03]). However, as the interventions included in this study were not directly targeting depression, a lower effect estimate is not unexpected compared to interventions directly targeting depression.

The finding that interventions targeting alcohol use can improve depression symptoms could be explained by several possible mechanisms, underpinned by research indicating that excessive alcohol use precedes more severe depression symptoms [14, 31]. First, evidence suggests that the metabolic and neurophysiological, and circadian rhythm changes which result from alcohol use increase the risk of depression [2]. Therefore, interventions targeting alcohol use may improve depression through these biological mechanisms. Second, hangovers which are result of excessive alcohol use can contribute to a cycle of feeling unwell, tired, lacking in concentration, guilty, anxious, and low mood [34], and thus interventions which reduce excessive alcohol use could help break this cycle. Third, excessive alcohol use can cause relationship difficulties, employment problems, impaired academic attainment, sexual and memory problems, as well as increasing the risk of accidental injury [22]. Thus, it is possible that if relationships, work, study, and other areas of life improve because of reducing alcohol use, depression symptoms may also improve. Fourth, an experience of success through reducing alcohol use could improve mood. Finally, it is also possible that some of these treatments could have a direct effect on mood independent of any effect on alcohol use. Future research should examine theory-derived potential mechanisms of change to improve our understanding of how interventions targeting excessive alcohol use in young people appear to achieve improvements in depressed mood.

### Study limitations

All studies eligible for inclusion in the meta-analysis were of University age participants, meaning that the results address a narrower age range than the WHO definition of young person as 10-24 which was used in this study’s research question. Moreover All studies included in the meta-analysis were conducted in University settings, thus limiting their representativeness. Moreover, four of the five studies were conducted in the USA, and so findings may not be generalizable to other countries including low and middle-income countries. Furthermore, the populations included tended to be largely White (although studies reported race/ethnicity inconsistently, and in one study not at all). One study [12] was with young people who self-identified as American Indian/Alaska Natives, but data was not available for inclusion in the meta-analysis, despite attempts to contact the authors. Thus, more research is needed with young people from more diverse socioeconomic and cultural backgrounds.

A further limitation is that several intervention studies were identified that looked at young people with mixed substance abuse problems but did not report separate outcomes for those with excessive alcohol use only, meaning that these studies could not be included in the current review. Moreover, several adult studies were identified that included some participants aged 18-24 years, but data for these individuals were not disaggregated from the full sample and thus could not be included. Finally, none of the studies included were judged to be at low risk of bias. One of the principal reasons for this was that studies did not publish a pre-specified analysis plan. This is important to ensure that reported results were not selected from multiple analyses, and so future research would benefit from the publication of a pre-specified analysis plan.

### Study strengths

This review also has many strengths. Our searches were comprehensive, using broad search terms to maximise sensitivity, and reference lists of studies included were also checked. We screened over 6000 records, and each record at the full-text screening stage was double screened. All studies included in the meta-analysis assessed depression using well-validated scales, and the heterogeneity in the meta-analysis was 0%. Furthermore, although two studies included relatively short follow-up periods (one-month), a sensitivity analysis indicated that the findings still held when only studies with longer follow-up periods (12 and 16-months) were included. Data (SMD and its variance) was calculated from studies which provided data which was not in a form which could be directly used in the meta-analysis in line with guidelines from the Cochrane Collaboration Handbook [15]. Where authors did not provide sufficient data to allow these calculations, they were contacted directly to request such data.

### Implications for practice

Excessive alcohol use in young people is a major public health problem in high-income countries. Given that excessive alcohol use in young people predicts a range of poor short and long-term outcomes, and that comorbid excessive alcohol use and depression is associated with worse outcomes than either problem alone (e.g., lower global functioning, life satisfaction and increased suicide risk; [3]), relatively brief interventions for excessive alcohol use that also improve mood potentially offer considerable benefits. Moreover, in the light of the increasing evidence that a range of higher order executive functions are still developing during adolescence and early adulthood [29], and that excessive alcohol use during this period has negative psychological, neurological, physical and social consequences, current findings suggest that the provision of such interventions when excessive alcohol use is taking root is also likely to be helpful for addressing mood problems. These findings also highlight the potential benefits of taking a more integrated approach to substance use and mental health, areas which have traditionally been treated separately [8].

## Conclusions

The results of this systematic review and meta-analysis suggests that psychosocial interventions primarily targeting excessive alcohol use can reduce depression symptoms in young people. Given that comorbid excessive alcohol use and depression symptoms predicts poorer outcomes than either excessive alcohol use or depression alone [3], this suggests that relatively brief interventions for excessive alcohol use potentially offer considerable benefits. However, this finding should be interpreted with caution due to the relatively small effect size and as no studies included were at low risk of bias. More research is needed to generalise these findings to non-student populations and resource limited settings, such as low and middle-income countries. In addition, further research is required to elucidate mechanisms of change, as this could produce more targeted, streamlined interventions, and thereby improving both their efficacy and cost-effectiveness.

## Supplementary Information

**Additional file 1: Appendix 1A.** Example Search Strategy for APA PsycNET. **Appendix 1B.** Characteristics of Studies included in the Meta-Analysis. **Appendix 1C.** Characteristics of Studies included in the Narrative Synthesis. **Appendix 1D.** Risk of Bias Within Studies. **Appendix 1E.** PRISMA Checklist. **Appendix 1F.** Full text studies excluded, with reasons for exclusion.

## Data Availability

The datasets generated and/or analysed during the current study are available via the Open Science Framework Website, with a DOI, upon publication of the manuscript.
