# Supplementary material for: Do interventions principally targeting excessive alcohol use in young people improve depression symptoms?: a systematic review and meta-analysis
Source: BMC Psychiatry. 2022 Jun 21;22:417. doi: 10.1186/s12888-022-04006-x (PMC9214998; doi:10.1186/s12888-022-04006-x)
Supplement: Supplementary file 1 — Additional file 1: Appendix 1A. Example Search Strategy for APA PsycNET. Appendix 1B. Characteristics of Studies included in the Meta-Analysis. Appendix 1C. Characteristics of Studies included in the Narrative Synthesis. Appendix 1D. Risk of Bias Within Studies. Appendix 1E. PRISMA Checklist. Appendix 1F. Full text studies excluded, with reasons for exclusion. [file 12888_2022_4006_MOESM1_ESM.docx]

#### Appendix 1A: Example Search Strategy for APA PsycNET

1. Alcohol abuse. ab, kw, ti.
2. binge drinking.ab, kw, ti.
3. Alcoholism.ab, kw, ti.
4. hazardous drinking.ab, kw, ti.
5. excessive alcohol use.ab, kw, ti.
6. underage drinking.ab, kw, ti.
7. college drinking.ab, kw, ti.
8. alcohol*.ab, kw, ti.
9. problem*drinking.ab, kw, ti.
10. 1 or 2 or 3 or 4 or 5 or 6 or 7 or 8 or 9 or 10
11. psychological intervention.ab, kw, ti.
12. psychosocial intervention.ab, kw, ti.
13. psychotherapy.ab, kw, ti.
14. Motivational interview*.ab, kw, ti.
15. brief motivational intervention.ab, kw, ti.
16. cognitive behavio?ral therapy.ab, kw, ti.
17. family therapy.ab, kw, ti.
18. Mindfulness.ab, kw, ti.
19. acceptance and commitment therapy.ab, kw, ti.
20. dialectical behavio?r therapy.ab, kw, ti.
21. online intervention.ab, kw, ti.
22. 11 or 12 or 13 or 14 or 15 or 16 or 17 or 18 or 19 or 20 or 21
23. Youth.ab, kw, ti.
24. young people.ab, kw, ti.
25. Adolescen*.ab, kw, ti.
26. teenage*.ab, kw, ti.
27. college students.ab, kw, ti.
28. young adult.ab, kw, ti.
29. 23 or 24 or 25 or 26 or 27 or 28
30. Depress*.ab, kw, ti.
31. dysthym*.ab, kw, ti.
32. affect* (ti, kw)
33. mood.ab, kw, ti.
34. Anhedonia.ab, kw, ti.ab, kw, ti.
35. internali?ing symptoms.ab, kw, ti.
36. 30 or 31 or 32 or 33 or 34 or 35
37. random*.ab, kw, ti.
38. trial.ab, kw, ti.
39. control*.ab, kw, ti.
40. group*.ab, kw, ti.
41. placebo*.ab, kw, ti.
42. pseudo*.ab, kw, ti.
43. Non-randomised.ab, kw, ti.
44. Quasi-randomised.ab, kw, ti.
45. Intervention study.ab, kw, ti.
46. Intervention.ab, kw, ti.
47. 37 or 38 or 39 or 40 or 41 or 42 or 43 or 44 or 45 or 46
48. 10 and 22 and 29 and 36 and 47

#### Appendix 1B: Characteristics of Studies included in the Meta-Analysis

**Table 1**

*Characteristics of Studies included in the Meta-Analysis*

| First author | Year | Country and setting | Participants | Baseline depression score | Intervention | Comparison | Depression measure | Study design | Longest follow-up  (months) |
| --- | --- | --- | --- | --- | --- | --- | --- | --- | --- |
| Murphy | 2012 | USA,  University | Undergraduates (mean age = 18.5, SD = 0.71) who reported at least 2 heavy drinking episodes (≥5 drinks on one occasion) in the last month  Gender: 50% male 50% female  Ethnicity: 81.7% White/European American, 12.2% as Black/African American, 2.4% as Hispanic/Latino, 1.2% as Asian, and 1.2% as Native American (n = 82) | Depression SFAS at baseline (mean = 7.76; SD = 8.45); relaxation at baseline (mean = 6.97; SD = 6.19) | Brief motivational intervention plus substance free activity scheduling intervention | Brief motivational intervention plus relaxation training | Depression, Anxiety and Stress Scale (DASS) depression subscale (Mahmoud, Hall, & Staten, 2010) | Randomised controlled trial | 6 |
| Pengpid | 2013 | South Africa,  University | Students (mean age = 21.9, SD = 3.55) who were at-risk drinkers, defined as scoring ≥ 8 Alcohol Use Disorders Identification Test (AUDIT; Saunders, Aasland, Babor, & de la Fuente, 1993)  Gender: 87.3% male, 32.7% female  Ethnicity: not reported  (n=152) | Not reported | Brief counselling session on alcohol risk reduction | Feedback on alcohol screening and educational leaflet on responsible drinking | 10-item version of the Centres for epidemiologic Studies Depression Scale (CES-D Andresen, Malmgren, Carter & Patrick, 1994) | Randomised controlled trial | 12 |
| Geisner | 2015 | USA,  University | Undergraduates (mean age = 20.14, SD = 1.34) who reported drinking 4+ drinks for women or 5+ drinks for men on one occasion at least once in the last month, a problem drinking score of ≥8 on the AUDIT, and a depressed mood score of ≥ 14 on the Beck Depression Inventory II (BDI-II; Beck, Steer, & Brown, 1996)  Gender: 62% female, 38% male  Ethnicity: 59.7% White, 19.4% Asian or Pacific Islander, 1.2% Black/African American, 8.4% Multiracial, <1% Native American  (n = 339) | Gender differences in levels of depression at baseline:  females (mean = 23.51, SD = 7.94); males (mean = 22.57, SD = 7.25) | Web-based intervention using psychoeducation and personalised feedback | Assessment only | Depressive symptoms (measured by BDI-II) | Randomised controlled trial | 1 |
| Pedrelli | 2019 | USA,  University | Undergraduates (mean age = 19.9, SD = 1.4) with a Beck Depression Inventory (BDI; Beck, Ward, Mendelson, Mock, & Erbaugh, 1961) score of 10-30 (mild-moderate but not severe depressive symptoms), and at least one episode of heavy episodic drinking (5+ drinks for men and 4+ drinks for women in two hours)  Gender: 68.1% female, 31.9% male  Ethnicity: 54.2% White, 82.1% non-Hispanic  (n = 94) | BDI score at baseline (mean = 19.5, SD = 6.8) | CBT for depression plus brief motivational interviewing | CBT for depression | BDI (Beck et al., 1961) | Randomised controlled trial | 1 |
| Murphy | 2019 | USA,  University | Undergraduates (mean age = 18.77, SD = 1.06) who reported at least two heavy drinking episodes (≥5 drinks on one occasion) in the last month (n = 393)  Gender: 61% female, 49% male  Ethnicity: 85.2% White/European American, 10.9% Black/African American, 5.9% as Hispanic/Latino, 1.8% as Asian, and 1.8% as Native American | Depression Assessment-only at baseline (mean = 6.54, SD = 8.09); BMI+RT at baseline (mean = 8.01, SD = 9.16); BMI+SFAS at baseline (mean = 7.80; SD = 9.31) | Brief motivational intervention plus substance free activity scheduling intervention | Assessment only | Depression, Anxiety and Stress Scale (DASS) depression subscale | Randomised controlled trial | 16 |

#### Appendix 1C: Characteristics of Studies included in the Narrative Synthesis

**Table 2**

*Characteristics of Studies included in the Narrative Synthesis*

| First author, year, country and setting | Participants | Intervention | Comparison | Depression measure | Study design | Longest follow-up  (months) |
| --- | --- | --- | --- | --- | --- | --- |
| Fleming, 2009, Canada and the USA, College health clinics | College students (mean age = 20.9, SD = 2.25) who reported ≥8 heavy drinking episodes (≥5 units on one occasion) or ≥ 50 drinks in the last 28 days for men, or ≥ 6 heavy drinking episodes (≥ 4 units on one occasion) or ≥ 40 drinks in the last 28 days for women  Gender: 49.1% male, 50.9% female  Ethnicity/race: 90.7% non-Hispanic White  (n = 986) | Brief physician advice (including a motivational interviewing component) | Provided with a booklet on general health, and given 6- and 12-months follow-up phone calls (enhanced usual care) | Beck Depression Inventory for primary care (Beck et al., 1997) | Randomised controlled trial | 12 |
| Gilder, 2017, USA, American Indian/Alaska Natives (AI/AN) living on American Indian reservations | Participants aged 13-20 (no mean age or SD reported), who self-identified as AI/AN  and drank underage.  Gender: not reported  Ethnicity/race: 100% AI/AN  Level of education: not reported  (n = 69) | Motivational interviewing, adapted to incorporate both AI/AN and Western practices) | Psychoeducation | Student Self-Check (Dishion & Kavanagh, 2003) item 15 (Likert scale assessing the extent to which participants have felt “moody, withdrawn, sad or depressed” in the past month | Pilot randomised controlled trial | 24 (Mean) |
| Ngo, 2018, USA, Emergency department | Emergency department patients aged 14-20 years (mean age = 18.6, SD = 1.4) who obtained a positive screen on the AUDIT-C (≥ 3 for patients aged 14–17 years, or ≥4 for patients aged 18–20 years; Bush, Kivlahan, McDonell, Fihn, Bradley (1998)  Gender: 51.6% male, 40.4% female  Ethnicity/race: 79.4% White, 9.5% African American, 5.5%  Hispanic, 11.1% “other races”  Level of education: not reported  (n = 836) | Brief motivational interviewing intervention (therapist-delivered or computer-based) | Staff read through a brochure with participants, which included links to substance use and mental health services (enhanced usual care) | 7 item brief symptom inventory (Derogatis & Melisaratos, 1983) | Randomised controlled trial | 12 |

#### Appendix 1D: Risk of Bias Within Studies

**Table 3**

*Risk of Bias Within Studies*

| First Author/Year | Risk of bias arising from the randomisation process | Risk of bias due to deviations from the intended intervention | Risk of bias due to missing outcome data | Risk of bias due to measurement of the outcome | Risk of bias in selection of the reported result | Overall risk of bias |
| --- | --- | --- | --- | --- | --- | --- |
| Studies included in the meta-analysis |  |  |  |  |  |  |
| Murphy, 2012 | Some concerns | Some concerns | Low | Some concerns | Some concerns | Some concerns |
| Pengpid, 2013 | Low | Some concerns | Low | Some concerns | Some concerns | Some concerns |
| Geisner, 2015 | Low | Some concerns | Some concerns | Some concerns | Some concerns | Some concerns |
| Murphy, 2019 | Some concerns | Some concerns | Low | Low | Some concerns | Some concerns |
| Pedrelli, 2019 | Low | Some concerns | Low | Some concerns | Some concerns | Some concerns |
| Studies included in the narrative synthesis |  |  |  |  |  |  |
| Fleming, 2009 | Low | Low | Low | Low | Some concerns | Some concerns |
| Gilder, 2017 | Low | High | Some concerns | High | Some concerns | High risk |
| Ngo, 2018 | Low | Some concerns | Low | Some concerns | Some concerns | Some concerns |

**Appendix 1E: PRISMA Checklist^[[1]](#footnote-1)^**

| **Section and Topic** | **Item #** | **Checklist item** | **Location where item is reported** |
| --- | --- | --- | --- |
| **TITLE** | | |  |
| Title | 1 | Identify the report as a systematic review. |  |
| **ABSTRACT** | | |  |
| Abstract | 2 | See the PRISMA 2020 for Abstracts checklist. |  |
| **INTRODUCTION** | | |  |
| Rationale | 3 | Describe the rationale for the review in the context of existing knowledge. |  |
| Objectives | 4 | Provide an explicit statement of the objective(s) or question(s) the review addresses. |  |
| **METHODS** | | |  |
| Eligibility criteria | 5 | Specify the inclusion and exclusion criteria for the review and how studies were grouped for the syntheses. |  |
| Information sources | 6 | Specify all databases, registers, websites, organisations, reference lists and other sources searched or consulted to identify studies. Specify the date when each source was last searched or consulted. |  |
| Search strategy | 7 | Present the full search strategies for all databases, registers and websites, including any filters and limits used. |  |
| Selection process | 8 | Specify the methods used to decide whether a study met the inclusion criteria of the review, including how many reviewers screened each record and each report retrieved, whether they worked independently, and if applicable, details of automation tools used in the process. |  |
| Data collection process | 9 | Specify the methods used to collect data from reports, including how many reviewers collected data from each report, whether they worked independently, any processes for obtaining or confirming data from study investigators, and if applicable, details of automation tools used in the process. |  |
| Data items | 10a | List and define all outcomes for which data were sought. Specify whether all results that were compatible with each outcome domain in each study were sought (e.g., for all measures, time points, analyses), and if not, the methods used to decide which results to collect. |  |
|  | 10b | List and define all other variables for which data were sought (e.g., participant and intervention characteristics, funding sources). Describe any assumptions made about any missing or unclear information. |  |
| Study risk of bias assessment | 11 | Specify the methods used to assess risk of bias in the included studies, including details of the tool(s) used, how many reviewers assessed each study and whether they worked independently, and if applicable, details of automation tools used in the process. |  |
| Effect measures | 12 | Specify for each outcome the effect measure(s) (e.g., risk ratio, mean difference) used in the synthesis or presentation of results. |  |
| Synthesis methods | 13a | Describe the processes used to decide which studies were eligible for each synthesis (e.g., tabulating the study intervention characteristics and comparing against the planned groups for each synthesis (item #5)). |  |
|  | 13b | Describe any methods required to prepare the data for presentation or synthesis, such as handling of missing summary statistics, or data conversions. |  |
|  | 13c | Describe any methods used to tabulate or visually display results of individual studies and syntheses. |  |
|  | 13d | Describe any methods used to synthesize results and provide a rationale for the choice(s). If meta-analysis was performed, describe the model(s), method(s) to identify the presence and extent of statistical heterogeneity, and software package(s) used. |  |
|  | 13e | Describe any methods used to explore possible causes of heterogeneity among study results (e.g., subgroup analysis, meta-regression). |  |
|  | 13f | Describe any sensitivity analyses conducted to assess robustness of the synthesized results. |  |
| Reporting bias assessment | 14 | Describe any methods used to assess risk of bias due to missing results in a synthesis (arising from reporting biases). |  |
| Certainty assessment | 15 | Describe any methods used to assess certainty (or confidence) in the body of evidence for an outcome. |  |
| **RESULTS** | | |  |
| Study selection | 16a | Describe the results of the search and selection process, from the number of records identified in the search to the number of studies included in the review, ideally using a flow diagram. |  |
|  | 16b | Cite studies that might appear to meet the inclusion criteria, but which were excluded, and explain why they were excluded. |  |
| Study characteristics | 17 | Cite each included study and present its characteristics. |  |
| Risk of bias in studies | 18 | Present assessments of risk of bias for each included study. |  |
| Results of individual studies | 19 | For all outcomes, present, for each study: (a) summary statistics for each group (where appropriate) and (b) an effect estimates and its precision (e.g. confidence/credible interval), ideally using structured tables or plots. |  |
| Results of syntheses | 20a | For each synthesis, briefly summarise the characteristics and risk of bias among contributing studies. |  |
|  | 20b | Present results of all statistical syntheses conducted. If meta-analysis was done, present for each the summary estimate and its precision (e.g., confidence/credible interval) and measures of statistical heterogeneity. If comparing groups, describe the direction of the effect. |  |
|  | 20c | Present results of all investigations of possible causes of heterogeneity among study results. |  |
|  | 20d | Present results of all sensitivity analyses conducted to assess the robustness of the synthesized results. |  |
| Reporting biases | 21 | Present assessments of risk of bias due to missing results (arising from reporting biases) for each synthesis assessed. |  |
| Certainty of evidence | 22 | Present assessments of certainty (or confidence) in the body of evidence for each outcome assessed. |  |
| **DISCUSSION** | | |  |
| Discussion | 23a | Provide a general interpretation of the results in the context of other evidence. |  |
|  | 23b | Discuss any limitations of the evidence included in the review. |  |
|  | 23c | Discuss any limitations of the review processes used. |  |
|  | 23d | Discuss implications of the results for practice, policy, and future research. |  |
| **OTHER INFORMATION** | | |  |
| Registration and protocol | 24a | Provide registration information for the review, including register name and registration number, or state that the review was not registered. |  |
|  | 24b | Indicate where the review protocol can be accessed, or state that a protocol was not prepared. |  |
|  | 24c | Describe and explain any amendments to information provided at registration or in the protocol. |  |
| Support | 25 | Describe sources of financial or non-financial support for the review, and the role of the funders or sponsors in the review. |  |
| Competing interests | 26 | Declare any competing interests of review authors. |  |
| Availability of data, code and other materials | 27 | Report which of the following are publicly available and where they can be found template data collection forms; data extracted from included studies; data used for all analyses; analytic code; any other materials used in the review. |  |

*From:*  Page MJ, McKenzie JE, Bossuyt PM, Boutron I, Hoffmann TC, Mulrow CD, et al. The PRISMA 2020 statement: an updated guideline for reporting systematic reviews. BMJ 2021;372: n71. doi: 10.1136/bmj. n71

**Appendix 1F: Full text studies excluded, with reasons for exclusion**

| **Title** | **Authors** | **Published Year** | **Reason for exclusion** |
| --- | --- | --- | --- |
| "Evaluating the combination of a Brief Motivational Intervention plus Cognitive Behavioral Therapy for Depression and heavy episodic drinking in college students": Correction to Pedrelli et al. (2019). | Anonymous | 2020 | Preliminary results: complete results presented elsewhere. Duplicate. |
| A 9-month follow-up of a 3-month web-based alcohol treatment program using intensive asynchronous therapeutic support | Postel, MG. ter Huurne, ED. de Haan, HA. van der Palen, J. de Jong, CA | 2015 | Wrong patient population. |
| A behavioral economic intervention reduces drinking among college student drinkers with psychiatric symptoms | Dennhardt, AA. Martens, MP. Borsari, B. Witkiewitz, K. Murphy, JG | 2017 | Conference abstract-no results available |
| A behavioral economic supplement improves brief alcohol intervention outcomes | Murphy, JG. Dennhardt, AA. Borsari, B. Martens, MP. Witkiewitz, KA | 2016 | Wrong outcomes. |
| A brief behavioral economic intervention moderates the impact of self-regulation on alcohol use and problems | Soltis, KE. Dennhardt, AA. Martens, M. Murphy, JG | 2017 | Conference abstract-no results available |
| A brief motivational interview in a pediatric emergency department, plus 10-day telephone follow-up, increases attempts to quit drinking among youth and young adults who screen positive for problematic drinking | Bernstein, J; Heeren, T; Edward, E; Dorfman, D; Bliss, C; Winter, M; Bernstein, E | 2010 | Wrong outcomes. |
| A brief personalized feedback intervention integrating a motivational interviewing therapeutic style and dialectical behavioral therapy skills for depressed or anxious heavy drinking young adults. | Whiteside, Ursula | 2011 | Conference abstract-no results available |
| A DAILY PROCESS APPROACH TO EVALUATING 3 BRIEF MOTIVATION-BASED INTERVENTIONS TO REDUCE YOUNG ADULTS' ALCOHOL USE | Collins, R. L.; Vincent, P.; Dermen, K.; Vetter, C.; Wilson, S.; Smith, J. | 2010 | Conference abstract-no results available |
| A developmental-based motivational intervention to reduce alcohol and marijuana use among non-treatment-seeking young adults: a randomized controlled trial | Stein, Michael D. Caviness, Celeste M. Morse, Emily F. Grimone, Kristin R. Audet, Daniel. Herman, Debra S. Moitra, Ethan. Anderson, Bradley J. | 2018 | Wrong patient population. |
| A multilevel analysis examining treatment modality and dysphoria as predictors of college drinking following a brief alcohol treatment | King, SC; Hoffman, L; McChargue, DE | 2013 | Conference abstract-no results available |
| A randomised controlled trial of enhanced cognitive therapy and family education for youth depression, anxiety, and substance abuse | ACTRN12609000630213, | 2009 | Wrong intervention. |
| A randomized clinical trial of a brief, mailed intervention for depressed mood in a college student sample. | Geisner, Irene Markman | 2009 | Wrong intervention. |
| A randomized clinical trial of a targeted intervention to moderate alcohol use and alcohol-related problems in at-risk adolescents | Thush, C; Wiers, RW; Theunissen, N; Van den Bosch, J; Opdenacker, J; van Empelen, P; Moerbeek, M; Feron, FJ | 2007 | Wrong outcomes. |
| A randomized controlled trial of cognitive-behavioral treatment for depression versus relaxation training for alcohol-dependent individuals with elevated depressive symptoms | Brown, RA. Ramsey, SE. Kahler, CW. Palm, KM. Monti, PM. Abrams, D. Dubreuil, M. Gordon, A. Miller, IW | 2011 | Adult population. |
| A Randomized Controlled Trial of Structured Stepped-care Intervention for Psychiatric Comorbidity | NCT01941693, | 2013 | Adult population. |
| A randomized trial of an integrated cognitive behavioral treatment protocol for adolescents receiving home-based services for co-occurring disorders. | Wolff, Jennifer. Esposito-Smythers, Christianne. Frazier, Elisabeth. Stout, Robert. Gomez, Judy. Massing-Schaffer, Maya. Nestor, Bridget. Cheek, Shayna. Graves, Hannah. Yen, Shirley. Hunt, Jeffrey. Spirito, Anthony | 2020 | Study protocol. |
| A randomized trial of motivational interviewing for prevention of underage drinking in American Indian adolescents | Gilder, D. Geisler, J. Luna, J. Calac, D. Moore, R. Ehlers, C | 2017 | This publication has been withdrawn. |
| A recipe for good mental health: A pilot randomised controlled trial of a psychological wellbeing and substance use intervention targeting young chefs | Pidd, Ken. Roche, Ann. Fischer, Jane | 2015 | Adult population. |
| A small group approach to youth education about alcohol. | Kunkle-Miller, Carole. Blane, Howard T. | 1977 | Wrong study design. |
| Adapting a blended motivational interviewing and problem-solving intervention to address risky substance use amongst South Africans | Sorsdahl, Katherine. Myers, Bronwyn. Ward, Catherine L. Matzopoulos, Richard. Mtukushe, Bulelwa. Nicol, Andrew. Cuijpers, Pim. Stein, Dan J. | 2015 | Wrong patient population. |
| Addition of cue exposure to cognitive-behaviour therapy for alcohol misuse: a randomized trial with dysphoric drinkers | Kavanagh, DJ. Sitharthan, G. Young, RM. Sitharthan, T. Saunders, JB. Shockley, N. Giannopoulos, V | 2006 | Wrong patient population. |
| Affect regulation training (ART) for alcohol use disorders: development of a novel intervention for negative affect drinkers | Stasiewicz, PR. Bradizza, CM. Schlauch, RC. Coffey, SF. Gulliver, SB. Gudleski, GD. Bole, CW | 2013 | Adult population. |
| Alpha wave biofeedback training therapy in alcoholics | Passini, FT. Watson, CG. Dehnel, L. Herder, J. Watkins, B | 1977 | Adult population. |
| An effectiveness trial of group cognitive behavioral therapy for patients with persistent depressive symptoms in substance abuse treatment | Watkins, KE. Hunter, SB. Hepner, KA. Paddock, SM. de la Cruz, E. Zhou, AJ. Gilmore, J | 2011 | Adult population. |
| An evaluation of a brief motivational intervention among young ecstasy and cocaine users: no effect on substance and alcohol use outcomes | Marsden, John. Stillwell, Garry. Barlow, Helen. Boys, Annabel. Taylor, Colin. Hunt, Neil. Farrell, Michael | 2006 | Wrong outcomes. |
| An evaluation of the effectiveness of a psychological treatment for moderate to severe depression and harmful or dependent drinking in rural communities in Nepal | ISRCTN72875710, | 2014 | Adult population. |
| An evaluation of the effectiveness of delivery of Health Promotion Interventions to people with serious mental illness by their key workers | ISRCTN95265680, | 2006 | Adult population. |
| An online intervention for co-occurring depression and problematic alcohol use in young people: Primary outcomes from a randomized controlled trial. | Deady, Mark; Mills, Katherine L.; Teesson, Maree; Kay-Lambkin, Frances | 2016 | Wrong outcomes. |
| Anonymous Treatment on the Internet for Alcohol Dependence: a Randomised, Controlled Trial | NCT02377726, | 2015 | Adult population. |
| Behavioral and psychophysiological effects of a yoga intervention on high-risk adolescents: A randomized control trial. | Fishbein, Diana. Miller, Shari. Herman-Stahl, Mindy. Williams, Jason. Lavery, Bud. Markovitz, Lara. Kluckman, Marianne. Mosoriak, Greg. Johnson, Michelle | 2016 | Wrong patient population. |
| Brief Acceptance and Commitment Therapy for HIV-infected At-risk Drinkers | NCT03974061, | 2019 | Wrong patient population. |
| Brief alcohol counseling improves mental health functioning in veterans with alcohol misuse: results from a randomized trial | Cucciare, MA. Boden, MT. Weingardt, KR | 2013 | Adult population. |
| Brief Intervention for Families of Teens Treated in the Emergency Department for an Alcohol-Related Event | NCT00247221, | 2005 | Wrong outcomes. |
| Brief intervention for women with risky drinking and medical diagnoses: a randomized controlled trial | Chang, G. Fisher, ND. Hornstein, MD. Jones, JA. Hauke, SH. Niamkey, N. Briegleb, C. Orav, EJ | 2011 | Adult population. |
| Brief intervention to reduce problem drinking in college students with ADHD. | Vasko, John M.; Meinzer, Michael C.; Murphy, James G.; Oddo, Lauren E.; McCauley, Katherine L.; Rooney, Mary E.; Lejuez, Carl W.; Chronis-Tuscano, Andrea | 2019 | Wrong intervention. |
| Brief interventions to address substance use among patients presenting to emergency departments in resource poor settings: a cost-effectiveness analysis | Dwommoh, R. Sorsdahl, K. Myers, B. Asante, KP. Naledi, T. Stein, DJ. Cleary, S | 2018 | Adult population. |
| CAMH PARTNERs Integrated Care Study | NCT02345122, | 2015 | Wrong patient population. |
| Can people with substance misuse and depression benefit from brief psychological therapies? | ISRCTN26937594, | 2012 | Adult population. |
| Clinical Outcomes From a 10-Week Follow-Up Psychoeducational Program for Dual Diagnosis | Chilton, J. Crone, D. Tyson, P. J. | 2018 | Adult population. |
| Clinician-assisted computerised versus therapist-delivered treatment for depressive and addictive disorders: a randomised controlled trial | Kay-Lambkin, FJ. Baker, AL. Kelly, B. Lewin, TJ | 2011 | Wrong patient population. |
| Cognitive-behavioral therapy in depressed primary care patients with co-occurring problematic alcohol use: effect of telephone-administered vs. face-to-face treatment-a secondary analysis | Kalapatapu, RK. Ho, J. Cai, X. Vinogradov, S. Batki, SL. Mohr, DC | 2014 | Adult population. |
| Cognitive-behavioural therapy for substance use disorders in people with psychotic disorders: randomised controlled trial | Baker, A. Bucci, S. Lewin, TJ. Kay-Lambkin, F. Constable, PM. Carr, VJ | 2006 | Adult population. |
| Collaborative Management of Depression and Alcohol Misuse by General Practitioners and Correspondence-Based Programs | ACTRN12605000647639, | 2005 | Adult population. |
| Combined MI | Pedrelli, Paola. Borsari, Brian. Palm, Kathleen. Dalton, Elizabeth. Fava, Maurizio | 2013 | Wrong study design. |
| Combining Motivational Interviewing With Compliance Enhancement Therapy (MI-CET): Development and Preliminary Evaluation of a New, Manual-Guided Psychosocial Adjunct to Alcohol-Dependence Pharmacotherapy | Heffner, Jaimee L. Tran, Giao Q. Johnson, Candace S. Barrett, Suzan Winders. Blom, Thomas J. Thompson, Rachel D. Anthenelli, Robert M. | 2010 | Adult population. |
| Community-Based Integrated Treatment for Adolescents | NCT01667159, | 2012 | Study protocol. |
| Comorbid anxiety disorders and baseline medication regimens predict clinical outcomes in individuals with co-occurring bipolar disorder and alcohol dependence: results of a randomized controlled trial | Prisciandaro, JJ. Brown, DG. Brady, KT. Tolliver, BK | 2011 | Adult population. |
| Comorbidity of substance dependence and depression: role of life stress and self-efficacy in sustaining abstinence | Tate, SR. Wu, J. McQuaid, JR. Cummins, K. Shriver, C. Krenek, M. Brown, SA | 2008 | Adult population. |
| Comparison of a brief versus extended telephone delivered intervention for hazardous alcohol use among young people living with severe mental ill-health | ACTRN12619000559112, | 2019 | Study protocol. |
| Comparison of treatment modalities for adolescent offenders who participated in a substance abuse treatment program. | Pham, Stacie H. | 2011 | Wrong patient population. |
| Computer-delivered personalized feedback intervention for hazardous drinkers with elevated anxiety sensitivity: Study protocol for a randomized controlled trial | Paulus, Daniel J.; Gallagher, Matthew W.; Neighbors, Clayton; Zvolensky, Michael J. | 2020 | Study protocol. |
| Computer-Delivered PFI for Anxiety Sensitivity/Alcohol Intervention for Hazardous Drinkers With Elevated Anxiety Sensitivity | NCT03917875, | 2019 | Study protocol. |
| Cost-effectiveness analysis of four interventions for adolescents with a substance use disorder | French, Michael T. Zavala, Silvana K. McCollister, Kathryn E. Waldron, Holly B. Turner, Charles W. Ozechowski, Timothy J. | 2008 | Wrong outcomes. |
| Depression, Craving, and Substance Use Following a Randomized Trial of Mindfulness-Based Relapse Prevention | Witkiewitz, Katie. Bowen, Sarah | 2010 | Wrong patient population. |
| Depressive symptoms and heavy drinking in college students: How should wetreat them? | Pedrelli P. Borsari B. | 2015 | Preliminary results: complete results presented elsewhere. |
| Depressive symptoms as a moderator of college student response to computerized alcohol intervention | Miller, Mary Beth. Hall, Nicole. DiBello, Angelo M. Park, Chan Jeong. Freeman, Lindsey. Meier, Ellen. Leavens, Eleanor L. S. Leffingwell, Thad R. | 2020 | Wrong outcomes. |
| Developing socialmedia interventions for risky drinking among adolescents and emerging adults | Walton, MA. Bauermeister, J. Young, S. Blow, FC. Schneeberger, D. Bourque, C. Cunningham, RM. Bohnert, A. Bonar, E | 2018 | Wrong outcomes. |
| Do outcomes of cognitive-behaviour therapy for co-occurring alcohol misuse and depression differ for participants with symptoms of posttraumatic stress? | Bailey, KA. Baker, AL. McElduff, P. Kay-Lambkin, F. Kavanagh, DJ | 2019 | Adult population. |
| Do young people benefit from AA as much, and in the same ways, as adult aged 30+? A moderated multiple mediation analysis | Hoeppner, Bettina B.; Hoeppner, Susanne S.; Kelly, John F. | 2014 | Wrong patient population. |
| Does initial treatment focus influence outcomes for depressed substance abusers? | Drapkin, ML. Tate, SR. McQuaid, JR. Brown, SA | 2008 | Adult population. |
| Does providing a brief internet intervention for hazardous alcohol use to people seeking online help for depression reduce both alcohol use and depression symptoms among participants with these co-occurring disorders? Study protocol for a randomised cont | Cunningham, JA. Hendershot, CS. Kay-Lambkin, F. Neighbors, C. Griffiths, KM. Bennett, K. Bennett, A. Godinho, A. Schell, C | 2018 | Adult population. |
| Does providing a brief internet intervention for hazardous alcohol use to people seeking online help for depression reduce both alcohol use and depression symptoms among participants with these co-occurring disorders? Study protocol for a randomised contr | Cunningham, John A. Hendershot, Christian S. Kay-Lambkin, Frances. Neighbors, Clayton. Griffiths, Kathleen M. Bennett, Kylie. Bennett, Anthony. Godinho, Alexandra. Schell, Christina | 2018 | Adult population. |
| Does temperament moderate treatment response in adolescent substance use disorders? | Kaminer, Y. Burleson, JA | 2008 | Wrong outcomes. |
| Does the addition of integrated cognitive behaviour therapy and motivational interviewing improve the outcomes of standard care for young people with comorbid depression and substance misuse? | Hides, Leanne M. Elkins, Kathryn S. Scaffidi, Antonietta. Cotton, Sue M. Carroll, Steve. Lubman, Daniel I. | 2011 | Wrong patient population. |
| Does the inclusion of an emotional wellbeing intervention into an existing, web-based, health promotion programme improve public sector staffs ability to improve their health behaviours? Protocol for a feasibility study | ISRCTN50074817, | 2017 | Wrong patient population. |
| Don‚Äôt let alcohol be in control! Can we teach adolescents to gain control over alcohol-related impulses and thoughts? | ACTRN12616000077460, | 2016 | Wrong intervention. |
| Effect of short duration counselling for alcohol use which can cause harm in patients with mood problems: a randomised controlled trial | CTRI/2019/02/017461, | 2019 | Adult population. |
| Effectiveness and Costs of Internet-based Treatment for Harmful Alcohol Use and Face-to-face Treatment in Addiction Care | NCT02671019, | 2016 | Adult population. |
| Effectiveness of a self-help approach (Retraining in vivo) to reduce alcohol consumption | DRKS00015319, | 2018 | Adult population. |
| Effectiveness of brief alcohol interventions for general practice patients with problematic drinking behavior and comorbid anxiety or depressive disorders | Grothues, JM. Bischof, G. Reinhardt, S. Meyer, C. John, U. Rumpf, HJ | 2008 | Adult population. |
| Effectiveness of psychological treatments for depression and alcohol use disorder delivered by community-based counsellors: two pragmatic randomised controlled trials within primary healthcare in Nepal | Jordans, MJD. Luitel, NP. Garman, E. Kohrt, BA. Rathod, SD. Shrestha, P. Komproe, IH. Lund, C. Patel, V | 2019 | Adult population. |
| Effects of a brief ED-based alcohol and violence intervention on depressive symptoms. | Ranney, Megan L.; Goldstick, Jason; Eisman, Andria; Carter, Patrick M.; Walton, Maureen; Cunningham, Rebecca M. | 2017 | Wrong intervention. |
| Effects of a brief mindfulness intervention on negative affect and urge to drink among college student drinkers. | Vinci, C.; Peltier, M. R.; Shah, S.; Kinsaul, J.; Waldo, K.; McVay, M. A.; Copeland, A.L. | 2014 | Wrong outcomes. |
| Effects of a Single Lyric Analysis Intervention on Withdrawal and Craving With Inpatients on a Detoxification Unit: A Cluster-Randomized Effectiveness Study | Silverman, Michael J. | 2016 | Adult population. |
| Effects of AlcoholEdu for college on alcohol-related problems among freshmen: a randomized multicampus trial | Paschall, MJ. Antin, T. Ringwalt, CL. Saltz, RF | 2011 | Wrong intervention. |
| Effects of forgiveness therapy on anger, mood, and vulnerability to substance use among inpatient substance-dependent clients | Lin, WF. Mack, D. Enright, RD. Krahn, D. Baskin, TW | 2004 | Wrong patient population. |
| Effects of mindfulness on impulsivity of peope with alcohol use disorder | RBR-6c9njc, | 2019 | Study protocol. |
| Effects of Motivational Interviewing for Incarcerated Adolescents on Driving Under the Influence after Release. | Stein, L. A. R. Colby, Suzanne M. Barnett, Nancy P. Monti, Peter M. Golembeske, Charles. Lebeau-Craven, Rebecca | 2006 | Wrong patient population. |
| Efficacy of an internet-based self-help intervention to reduce co-occurring alcohol misuse and depression symptoms in adults: study protocol of a three-arm randomised controlled trial | Schaub, MP. Blankers, M. Lehr, D. Boss, L. Riper, H. Dekker, J. Goudriaan, AE. Maier, LJ. Haug, S. Amann, M. et al. | 2016 | Adult population. |
| Efficacy of an Online Self-Help Treatment for Comorbid Alcohol Misuse and Emotional Problems in Young Adults: Protocol for a Randomized Controlled Trial | Frohlich, Jona R. Rapinda, Karli K. Schaub, Michael P. Wenger, Andreas. Baumgartner, Christian. Johnson, Edward A. O'Connor, Roisin M. Vincent, Norah. Blankers, Matthijs. Ebert, David D. Hadjistavropoulos, Heather. Mackenzie, Corey S. Keough, Matthew T. | 2018 | Study protocol. |
| Efficacy of Grit Wellbeing program for individuals attending residential rehabilitation for substance use problems | ACTRN12617001451392, | 2017 | Adult population. |
| Efficacy of Treating Sexual Trauma in a Substance Abuse Residential Program for Women | Hemma, Gina. McNab, Amanda. Katz, Lori S. | 2018 | Adult population. |
| EMDR Versus Treatment As Usual in Patients With Substance Use Disorder | NCT03517592, | 2018 | Adult population. |
| Emotion Regulation Outcomes and Preliminary Feasibility Evidence From a Mindfulness Intervention for Adolescent Substance Use | Russell, Beth S. Hutchison, Morica. Fusco, Alaina | 2019 | Wrong patient population. |
| Emotion regulation treatment for alcohol use disorder improves both mental health and alcohol use disorder symptoms | Linn, BK. Stasiewicz, PR. Slosman, KS. Zhao, J. Ruszczyk, MU. Lucke, JF. Bradizza, CM | 2019 | Adult population. |
| En voz de la experiencia: Estrategias de enfrentamiento en adolescentes que concluyeron un programa de intervenci√≥n breve en consumo de alcohol. [In voice of experience: Coping strategies in adolescents who completed a brief intervention program in alcoho | Mart√≠nez, Kalina Isela Mart√≠nez. Mel√©ndez, Adriana B√°rcenas. Trejo, Ayme Yolanda Pacheco | 2012 | Article not available in English. |
| Enhancing residential substance abuse treatment with computer interventions | ACTRN12611000618954, | 2011 | Wrong patient population. |
| Evaluating a Brief, Internet-Based Intervention for Co-Occurring Depression and Problematic Alcohol Use in Young People: Protocol for a Randomized Controlled Trial | Deady, Mark. Teesson, Maree. Kay-Lambkin, Frances. Mills, Katherine L. | 2014 | Preliminary results: complete results presented elsewhere. |
| Evaluating the Brief Alcohol Screening for College Students (BASICS) in small group settings for mandated college students engaged in high-risk drinking. | Hill, LaMisha N. | 2014 | Wrong outcomes. |
| Evaluating the combination of a Brief Motivational Intervention plus Cognitive Behavioral Therapy for Depression and heavy episodic drinking in college students. | Pedrelli, Paola. Borsari, Brian. Merrill, Jennifer E. Fisher, Lauren B. Nyer, Maren. Shapero, Benjamin G. Farabaugh, Amy. Hayden, Emma R. Levine, M. Taylor. Fava, Maurizio. Weiss, Roger D. | 2020 | Duplicate. |
| Evaluation of an integrated group cognitive-behavioral treatment for comorbid mood, anxiety, and substance use disorders: A pilot study | Milosevic, Irena. Chudzik, Susan M. Boyd, Susan. McCabe, Randi E. | 2017 | Adult population. |
| Evaluation of cognitive behavioral therapy/motivational enhancement therapy (CBT/MET) in a treatment trial of comorbid MDD/AUD adolescents. | Cornelius, Jack R.; Douaihy, Antoine; Bukstein, Oscar G.; Daley, Dennis C.; Wood, Scott D.; Kelly, Thomas M.; Salloum, Ihsan M. | 2011 | Wrong intervention. |
| Examination of trait impulsivity on the response to a brief mindfulness intervention among college student drinkers. | Vinci, Christine; Peltier, MacKenzie; Waldo, Krystal; Kinsaul, Jessica; Shah, Sonia; Coffey, Scott F.; Copeland, Amy L. | 2016 | Wrong outcomes. |
| Examining the efficacy of a brief group protective behavioral strategies skills training alcohol intervention with college women. | Kenney, Shannon R. Napper, Lucy E. LaBrie, Joseph W. Martens, Matthew P. | 2014 | Wrong outcomes. |
| Exercise as an intervention for sedentary hazardous drinking college students: A pilot study | Weinstock, Jeremiah. Capizzi, Jeffrey. Weber, Stefanie M. Pescatello, Linda S. Petry, Nancy M. | 2014 | Wrong intervention. |
| Exploring treatment attendance and its relationship to outcome in a randomized controlled trial of treatment for alcohol problems: secondary analysis of the UK Alcohol Treatment Trial (UKATT) | Dale, V. Coulton, S. Godfrey, C. Copello, A. Hodgson, R. Heather, N. Orford, J. Raistrick, D. Slegg, G. Tober, G | 2011 | Adult population. |
| Facing up to binge drinking: reducing binge drinking in adolescent males | Dempster, M. Newell, G. Cowan, G. Marley, J | 2006 | Wrong outcomes. |
| Family intervention for co-occurring substance use and severe psychiatric disorders: participant characteristics and correlates of initial engagement and more extended exposure in a randomized controlled trial | Mueser, KT. Glynn, SM. Cather, C. Zarate, R. Fox, L. Feldman, J. Wolfe, R. Clark, RE | 2009 | Adult population. |
| From design considerations to dissemination: adapting addiction treatment for diverse populations | Lee, CS. Rosales, RA | 2019 | Adult population. |
| From the neurobiological basis of comorbid alcohol dependence and depression to psychological treatment strategies: study protocol of a randomized controlled trial | Becker, A. Ehret, AM. Kirsch, P | 2017 | Adult population. |
| FullFix: a pilot trial of a telephone delivered transdiagnostic intervention for comorbid substance and mental health problems in young people | ACTRN12618001563257, | 2018 | Wrong intervention. |
| Gender and depression moderate response to brief motivational intervention for alcohol misuse among college students. | Merrill, Jennifer E. Reid, Allecia E. Carey, Michael P. Carey, Kate B. | 2014 | Wrong outcomes. |
| Goal Setting for Health Behavior and Psychosocial Issues in Primary Care | NCT01825746, | 2013 | Adult population. |
| Group interpersonal psychotherapy (IPT) for incarcerated women with comorbid substance use and major depressive disorders | Johnson, J. Zlotnick, C | 2011 | Conference abstract-no results available |
| Guided and Unguided Internet Treatment for Problematic Alcohol Use | NCT02384304, | 2015 | Adult population. |
| Guided and Unguided Internet-Based Treatment for Problematic Alcohol Use - A Randomized Controlled Pilot Trial | Sundstrom, Christopher. Gajecki, Mikael. Johansson, Magnus. Blankers, Matthijs. Sinadinovic, Kristina. Stenlund-Gens, Erik. Berman, Anne H. | 2016 | Wrong patient population. |
| Hospital-based adolescent substance abuse treatment: Comorbidity, outcomes, and gender | Rivers, S. M.; Greenbaum, R. L.; Goldberg, E. | 2001 | Wrong patient population. |
| Imaginal retraining reduces alcohol craving in problem drinkers: a randomized controlled trial | Moritz, S. Paulus, AM. Hottenrott, B. Weierstall, R. Gallinat, J. Kuhn, S | 2019 | Adult population. |
| Impact of Art Therapy on Alexithymia in People With Alcohol Use Disorder | NCT04206930, | 2019 | Adult population. |
| Implementation and effectiveness of an early intervention program (QuikFix) for young people experiencing alcohol and other drug-related harm | ACTRN12618001010280, | 2018 | Wrong outcomes. |
| Individual and family motivational interventions for alcohol-positive adolescents treated in an emergency department: results of a randomized clinical trial | Spirito, A. Sindelar-Manning, H. Colby, SM. Barnett, NP. Lewander, W. Rohsenow, DJ. Monti, PM | 2011 | Wrong outcomes. |
| Individual versus group female-specific cognitive behavior therapy for alcohol use disorder | Epstein, EE. McCrady, BS. Hallgren, KA. Gaba, A. Cook, S. Jensen, N. Hildebrandt, T. Holzhauer, CG. Litt, MD | 2018 | Adult population. |
| Individual versusgroup female-specific cognitive behavior therapy for alcohol use disorder | Epstein, EE. McCrady, BS. Hallgren, KA | 2018 | Adult population. |
| Individually tailored E-health interventions for primary care patients with problematic alcohol use and co-occurring depressive symptoms: phase IIa - proof of concept study | DRKS00011635, | 2017 | Adult population. |
| Initial RCT of a distress tolerance treatment for individuals with substance use disorders | Bornovalova, MA. Gratz, KL. Daughters, SB. Hunt, ED. Lejuez, CW | 2012 | Adult population. |
| INTEGRATE: an integrated treatment for young people with psychological distress | ACTRN12619001522101, | 2019 | Study protocol. |
| Integrated care for comorbid alcohol dependence and anxiety and/or depressive disorder: study protocol for an assessor-blind, randomized controlled trial | Morley, KC. Baillie, A. Sannibale, C. Teesson, M. Haber, PS | 2013 | Adult population. |
| Integrated Treatment at the First Stage: increasing Motivation for Alcohol Patients with Comorbid Disorders during Inpatient Detoxification | Ostergaard, M. Jatzkowski, L. Seitz, R. Speidel, S. Weber, T. L√ºbke, N. H√∂cker, W. Odenwald, M | 2018 | Adult population. |
| Integrating Motivational Interviewing With Cognitive-behavioral Therapy | NCT04254120, | 2020 | Adult population. |
| Interactive Voice Response for Relapse Prevention Following Cognitive-Behavioral Therapy for Alcohol Use Disorders: A Pilot Study | Rose, Gail L. Skelly, Joan M. Badger, Gary J. Naylor, Magdalena R. Helzer, John E. | 2012 | Adult population. |
| Interactive voice response with feedback intervention in outpatient treatment of substance use problems in adolescents and young adults: A randomized controlled trial. | Andersson, Claes. √ñjehagen, Agneta. Olsson, Martin O. Br√•dvik, Louise. H√•kansson, Anders | 2017 | Wrong patient population. |
| Internet Based Cognitive Behavior Treatment for Alcohol Use Disorders | NCT02645721, | 2016 | Adult population. |
| Internet-based attentional bias modification training as add-on to regular treatment in alcohol and cannabis dependent outpatients: a study protocol of a randomized control trial | Heitmann, J. van Hemel-Ruiter, ME. Vermeulen, KM. Ostafin, BD. MacLeod, C. Wiers, RW. DeFuentes-Merillas, L. Fledderus, M. Markus, W. de Jong, PJ | 2017 | Wrong patient population. |
| Internet-Based Relapse Prevention vs Face to Face Therapy at an Employee Assistance Program | NCT02014779, | 2013 | Adult population. |
| Internet-based vs Face-to-face Treatment for Alcohol Dependence | NCT02888002, | 2016 | Adult population. |
| Interpersonal group psychotherapy for comorbid alcohol dependence and non-psychotic psychiatric disorders | Malat, Jan. Leszcz, Molyn. Negrete, Juan Carlos. Turner, Nigel. Collins, Jane. Liu, Eleanor. Toneatto, Tony | 2008 | Adult population. |
| Interventions on medical students' psychological health: A meta-analysis | Yusoff, Muhamad Saiful Bahri | 2014 | Not primary research |
| Is brief motivational intervention effective in reducing alcohol use among young men voluntarily receiving it? A randomized controlled trial | Gaume, J. Gmel, G. Faouzi, M. Bertholet, N. Daeppen, JB | 2011 | Wrong outcomes. |
| Is specialised, integrated treatment for alcohol use disorder and comorbid anxiety and/or depression better than treatment as usual? | Morley, KC. Baillie, A. Leung, S. Sannibale, C. Teesson, M. Haber, PS | 2015 | Adult population. |
| Is Specialized Integrated Treatment for Comorbid Anxiety, Depression and Alcohol Dependence Better than Treatment as Usual in a Public Hospital Setting? | Morley, KC. Baillie, A. Leung, S. Sannibale, C. Teesson, M. Haber, PS | 2016 | Adult population. |
| Is treatment of alcohol dependence equally effective when carried out in primary care as in a specialized alcohol dependence clinic? | ISRCTN84490505, | 2013 | Adult population. |
| It's worth a try: the treatment experiences of rural and Urban participants in a randomized controlled trial of computerized psychological treatment for comorbid depression and alcohol/other drug use | Kay-Lambkin, FJ. Baker, AL. Kelly, BJ. Lewin, TJ | 2012 | Adult population. |
| Lithium treatment of depressed and nondepressed alcoholics | Dorus, W. Ostrow, DG. Anton, R. Cushman, P. Collins, JF. Schaefer, M. Charles, HL. Desai, P. Hayashida, M. Malkerneker, U | 1989 | Adult population. |
| Long-term effects of brief substance use interventions for mandated college students: sleeper effects of an in-person personal feedback intervention | White, HR. Mun, EY. Pugh, L. Morgan, TJ | 2007 | Wrong outcomes. |
| LONGER-TERM EFFECTIVENESS OF CBT IN TREATMENT OF COMORBID AUD/MDD ADOLESCENTS. | Cornelius, Jack R.; Douaihy, Antoine B.; Kirisci, Levent; Daley, Dennis C. | 2013 | Wrong intervention. |
| Mailed treatment to augment primary care for alcohol disorders: a randomised controlled trial | Kavanagh, D. Connolly, JM | 2009 | Adult population. |
| Major depression and treatment response in adolescents with ADHD and substance use disorder | Warden, D. Riggs, PD. Min, SJ. Mikulich-Gilbertson, SK. Tamm, L. Trello-Rishel, K. Winhusen, T | 2012 | Wrong patient population. |
| Mechanisms of behavior change in alcoholics anonymous: does Alcoholics Anonymous lead to better alcohol use outcomes by reducing depression symptoms? | Kelly, JF. Stout, RL. Magill, M. Tonigan, JS. Pagano, ME | 2010 | Adult population. |
| Medication and Counseling for Controlled Drinking | NCT01115894, | 2010 | Adult population. |
| Medication and Counseling for Controlled Drinking (Project SMART) | NCT00444418, | 2007 | Adult population. |
| Mindfulness-Based Relapse Prevention for Substance Use Disorders: Effects on Cardiac Vagal Control and Craving Under Stress | Carroll, Haley. Lustyk, M. Kathleen B. | 2018 | Adult population. |
| Moderators of Brief Motivation-Enhancing Treatments for Alcohol-Positive Adolescents Presenting to the Emergency Department | Becker, Sara J. Jones, Richard N. Hernandez, Lynn. Graves, Hannah R. Spirito, Anthony | 2016 | Wrong outcomes. |
| Motivational and Cognitive Intervention for College Drinkers | NCT01204229, | 2010 | Study protocol. |
| Motivational Interventions for Heavy Drinking College Students: Examining the Role of Discrepancy-Related Psychological Processes. | McNally, Abigail M. Palfai, Tibor P. Kahler, Christopher W. | 2005 | Wrong outcomes. |
| Motivational interviewing does not affect risk drinking among young women: A randomised, controlled intervention study in Swedish youth health centres. | Palm, Anna. Olofsson, Niclas. Danielsson, Ingela. Skalkidou, Alkistis. Wennberg, Peter. H√∂gberg, Ulf | 2016 | Wrong outcomes. |
| Motivational interviewing for incarcerated adolescents: Effects of depressive symptoms on reducing alcohol and marijuana use after release. | Stein, L. A. R. Lebeau, Rebecca. Colby, Uzanne M. Barnett, Nancy P. Golembeske, Charles. Monti, Peter M. | 2011 | Wrong patient population. |
| Motivational Interviewing to Reduce Substance Use Among Depression Patients | NCT02420561, | 2015 | Adult population. |
| Motivational interviewing to reduce substance-related consequences: Effects for incarcerated adolescents with depressed mood. | Stein, L. A. R. Clair, Mary. Lebeau, Rebecca. Colby, Suzanne M. Barnett, Nancy P. Golembeske, Charles. Monti, Peter M. | 2011 | Wrong patient population. |
| Multidimensional Family Therapy for Young Adolescent Substance Abuse: Twelve-Month Outcomes of a Randomized Controlled Trial | Liddle, Howard A. Rowe, Cynthia L. Dakof, Gayle A. Henderson, Craig E. Greenbaum, Paul E. | 2009 | Wrong outcomes. |
| Mutual help groups, perceived status benefits, and well-being: a test with adult children of alcoholics with personal substance abuse problems | Kingree, JB. Thompson, M | 2000 | Adult population. |
| Non-specialist health worker interventions for the care of mental, neurological and substance-abuse disorders in low- and middle-income countries | van Ginneken, Nadja. Tharyan, Prathap. Lewin, Simon. Rao, Girish N. Meera, S. M. Pian, Jessica. Chandrashekar, Sudha. Patel, Vikram | 2013 | Not primary research |
| Novel treatments for alcohol dependence: a randomized controlled trial of structured stepped-care intervention for psychiatric comorbidity | ACTRN12607000039482, | 2007 | Adult population. |
| Outcome of CRA with Homeless Adolescents: preliminary findings | Slesnick, N. Prestopnik, JL. Meyers, RJ | 2005 | Wrong patient population. |
| Parents as interventionists: Addressing adolescent substance use | Botzet, Andria M. Dittel, Christine. Birkeland, Robyn. Lee, Susanne. Grabowski, John. Winters, Ken C. | 2019 | Wrong outcomes. |
| PAUSE: The Development and Implementation of a Novel Brief Intervention Program Targeting Cannabis and Alcohol Use Among University Students | Halladay, Jillian. Fein, Allan. MacKillop, James. Munn, Catharine | 2018 | Wrong patient population. |
| Pediatrician and Behavioral Clinician-Delivered Screening, Brief Intervention and Referral to Treatment: Substance Use and Depression Outcomes | Sterling, Stacy. Kline-Simon, Andrea H. Weisner, Constance. Jones, Ashley. Satre, Derek D. | 2018 | Wrong patient population. |
| Physical activity as treatment for alcohol use disorders (FitForChange): study protocol for a randomized controlled trial | Hallgren, M. Andersson, V. Ekblom, O. Andreasson, S | 2018 | Adult population. |
| Pilot study of interpersonal psychotherapy versus supportive psychotherapy for dysthymic patients with secondary alcohol abuse or dependence | Markowitz, JC. Kocsis, JH. Christos, P. Bleiberg, K. Carlin, A | 2008 | Adult population. |
| Practitioner Review: Evidence-based practice guidelines on alcohol and drug misuse among adolescents: a systematic review | Bekkering, G. E. Aertgeerts, B. Asueta-Lorente, J.-F. Autrique, M. Goossens, M. Smets, K. van Bussel, J. C. H. Vanderplasschen, W. Van Royen, P. Hannes, K. | 2014 | Not primary research |
| Pragmatic randomised controlled trial to evaluate the effectiveness and cost effectiveness of a multi-component intervention to reduce substance use and risk-taking behaviour in adolescents involved in the criminal justice system: A trial protocol (RISKIT | Coulton, Simon. Stockdale, Kelly. Marchand, Catherine. Hendrie, Nadine. Billings, Jenny. Boniface, Sadie. Butler, Steve. Deluca, Paolo. Drummond, Colin. Newbury-Birch, Dorothy. Pellatt-Higgins, Tracy. Stevens, Alex. Sutherland, Alex. Wilson, Ed | 2017 | Study protocol. |
| Predictors of positive drinking outcomes among youth receiving an alcohol brief intervention in the emergency department | Davis, AK. Arterberry, BJ. Bonar, EE. Chermack, ST. Blow, FC. Cunningham, RM. Walton, MA | 2018 | Wrong outcomes. |
| Process variables predicting changes in adolescent alcohol consumption and mental health symptoms following personality-targeted interventions | O'Leary-Barrett, M. Pihl, RO. Conrod, PJ | 2017 | Wrong intervention. |
| Profiles of drug use behavior change for adolescents in treatment | Waldron, HB; Turner, CW; Ozechowski, TJ | 2005 | Wrong outcomes. |
| Project Motion, A Study of Motivational Interviewing to Reduce Heavy or Problematic Drinking | NCT00665249, | 2008 | Adult population. |
| Protocol for the Inroads Study: A Randomized Controlled Trial of an Internet-Delivered, Cognitive Behavioral Therapy-Based Early Intervention to Reduce Anxiety and Hazardous Alcohol Use Among Young People | Stapinski, Lexine A. Prior, Katrina. Newton, Nicola C. Deady, Mark. Kelly, Erin. Lees, Briana. Teesson, Maree. Baillie, Andrew J. | 2019 | Study protocol. |
| Psychophysiological and self-reported emotional responding in alcohol-dependent college students: the impact of brief acceptance/mindfulness instruction | Vernig, PM. Orsillo, SM | 2009 | Wrong outcomes. |
| Psychotherapies for adolescent substance abusers - A pilot study | Kaminer, Y. Burleson, J. A. Blitz, C. Sussman, J. Rounsaville, B. J. | 1998 | Wrong patient population. |
| QuikFix: enhanced motivational interviewing interventions for youth substance use | Hides, Leanne. Wilson, Hollie. Quinn, Catherine. Sanders, Davina | 2016 | Wrong study design. |
| Randomised control trial to evaluating the effectiveness of the Hello Sunday Morning "Daybreak" program in reducing alcohol use by adults | ACTRN12618000010291, | 2018 | Adult population. |
| Randomised controlled feasibility trial of a web-based alcohol treatment programme | ISRCTN31789096, | 2014 | Adult population. |
| Randomized controlled trial of cognitive-behavioural therapy for coexisting depression and alcohol problems: short-term outcome | Baker, AL; Kavanagh, DJ; Kay-Lambkin, FJ; Hunt, SA; Lewin, TJ; Carr, VJ; Connolly, J | 2010 | Wrong patient population. |
| Randomized controlled trial of MICBT for co-existing alcohol misuse and depression: outcomes to 36-months | Baker, AL. Kavanagh, DJ. Kay-Lambkin, FJ. Hunt, SA. Lewin, TJ. Carr, VJ. McElduff, P | 2014 | Wrong patient population. |
| Randomized controlled trial on the effects of a skills-based workshop on medical students' management of problem drinking and alcohol dependence. | Kahan, Meldon. Wilson, Lynn. Midmer, Deana. Borsoi, Diane. Martin, Dawn | 2003 | Wrong intervention. |
| Randomized trial comparing computer-delivered and face-to-face personalized feedback interventions for high-risk drinking among college students | Wagener, TL. Leffingwell, TR. Mignogna, J. Mignogna, MR. Weaver, CC. Cooney, NJ. Claborn, KR | 2012 | Wrong outcomes. |
| Randomized Trial of Family Therapy Versus Nonfamily Treatment for Adolescent Behavior Problems in Usual Care | Hogue, Aaron. Dauber, Sarah. Henderson, Craig E. Bobek, Molly. Johnson, Candace. Lichvar, Emily. Morgenstern, Jon | 2015 | Wrong intervention. |
| Readiness to change is a predictor of reduced substance use involvement: findings from a randomized controlled trial of patients attending South African emergency departments | Myers, B. Stein, DJ. Sorsdahl, K | 2017 | Adult population. |
| Readiness to change is a predictor of reduced substance use involvement: findings from a randomized controlled trial of patients attending South African emergency departments | Myers, B. van der Westhuizen, C. Naledi, T. Stein, DJ. Sorsdahl, K | 2016 | Adult population. |
| Ready-to-Change: a telephone-based intervention for alcohol misuse | ACTRN12618000828224, | 2018 | Adult population. |
| Reducing Alcohol Use in Depressed Patients | NCT00183079, | 2005 | Wrong patient population. |
| Reducing Hazardous Alcohol Use & Human Immunodeficiency Virus (HIV) Viral Load | NCT02720237, | 2016 | Wrong patient population. |
| Running as treatment for hospitalized alcoholics: an experimental approach | Weber, A | 1984 | Wrong intervention. |
| Sertraline and cognitive behavioral therapy for depressed alcoholics: results of a placebo-controlled trial | Moak, DH. Anton, RF. Latham, PK. Voronin, KE. Waid, RL. Durazo-Arvizu, R | 2003 | Adult population. |
| Six-months outcomes of a randomised trial of supportive text messaging for depression and comorbid alcohol use disorder | Agyapong, VI. McLoughlin, DM. Farren, CK | 2013 | Adult population. |
| Sleep and aggression in substance-abusing adolescents: Results from an integrative behavioral sleep-treatment pilot program | Haynes, P. L. Bootzin, R. R. Smith, L. Cousins, J. Cameron, M. Stevens, S. | 2006 | Wrong intervention. |
| Smartphone-assisted abstinence maintenance after alcohol withdrawal | DRKS00017700, | 2019 | Adult population. |
| Stress management training as a prevention program for heavy social drinkers: Cognitions, affect, drinking, and individual differences. | Rohsenow, Damaris J.; Smith, Ronald E.; Johnson, Sandra | 1985 | Wrong intervention. |
| Study Comparing Two Types of Psychotherapy for Treating Depression and Substance Abuse | NCT00108407, | 2005 | Adult population. |
| Study protocol: a randomized controlled trial of a computer-based depression and substance abuse intervention for people attending residential substance abuse treatment | Kelly, PJ. Kay-Lambkin, FJ. Baker, AL. Deane, FP. Brooks, AC. Mitchell, A. Marshall, S. Whittington, M. Dingle, GA | 2012 | Adult population. |
| Substance use outcomes for mindfulness-based relapse prevention are partially mediated by reductions in stress: Results from a randomized trial | Davis, Jordan P. Berry, Daniel. Dumas, Tara M. Ritter, Ellen. Smith, Douglas C. Menard, Christopher. Roberts, Brent W. | 2018 | Wrong patient population. |
| Supportive Text Messages to Reduce Mood Symptoms and Problem Drinking- Randomised Controlled Pilot Trials | NCT02327858, | 2014 | Adult population. |
| Supportive text messaging for depression and comorbid alcohol use disorder: single-blind randomised trial | Agyapong, VI. Ahern, S. McLoughlin, DM. Farren, CK | 2012 | Adult population. |
| Temporal pathways of change in two randomized controlled trials for depression and harmful drinking in Goa, India | Singla, DR. Hollon, SD. Velleman, R. Weobong, B. Nadkarni, A. Fairburn, CG. Bhat, B. Gurav, M. Anand, A. McCambridge, J. et al. | 2020 | Adult population. |
| Testing the Efficacy of an Online Integrated Treatment for Comorbid Alcohol Misuse and Emotional Problems | NCT03406039, | 2018 | Adult population. |
| The DAISI Project ‚Äì Depression & Alcohol Integrated & Single-focused Interventions | ACTRN12607000057482, | 2007 | Wrong patient population. |
| The effect of assertive continuing care on continuing care linkage, adherence and abstinence following residential treatment for adolescents with substance use disorders | Godley, Mark D. Godley, Susan H. Dennis, Michael L. Funk, Rodney R. Passetti, Lora L. | 2007 | Wrong outcomes. |
| The effect of telephone-based intervention (TBI) in alcohol abusers: a pilot study | Wongpakaran, T. Petcharaj, K. Wongpakaran, N. Sombatmai, S. Boripuntakul, T. Intarakamhaeng, D. Wannarit, K | 2011 | Adult population. |
| The effectiveness and cost-effectiveness of lay counsellor-delivered psychological treatments for harmful and dependent drinking and moderate to severe depression in primary care in India: PREMIUM study protocol for randomized controlled trials | Patel, V. Weobong, B. Nadkarni, A. Weiss, HA. Anand, A. Naik, S. Bhat, B. Pereira, J. Araya, R. Dimidjian, S. et al. | 2014 | Adult population. |
| The effectiveness of integrated treatment in patients with substance use disorders co-occurring with anxiety and/or depression - a group randomized trial | Wusthoff, LE. Waal, H. Grawe, RW | 2014 | Adult population. |
| The efficacy of a blended motivational interviewing and problem solving therapy intervention to reduce substance use among patients presenting for emergency services in South Africa: A randomized controlled trial | Sorsdahl, K. Stein, D. J. Corrigall, J. Cuijpers, P. Smits, N. Naledi, T. Myers, B. | 2015 | Wrong patient population. |
| The efficacy of a brief intervention to reduce alcohol misuse in patients with HIV in South Africa: study protocol for a randomized controlled trial | Huis In 't Veld, D. Skaal, L. Peltzer, K. Colebunders, R. Ndimande, JV. Pengpid, S | 2012 | Adult population. |
| The Efficacy of a Standalone Protective Behavioral Strategies Intervention for Students Accessing Mental Health Services | LaBrie, Joseph W. Napper, Lucy E. Grimaldi, Elizabeth M. Kenney, Shannon R. Lac, Andrew | 2015 | Wrong outcomes. |
| The impact of youth internalising and externalising symptom severity on the effectiveness of brief personality-targeted interventions for substance misuse: a cluster randomised trial | Perrier-M√©nard, E. Castellanos-Ryan, N. O'Leary-Barrett, M. Girard, A. Conrod, PJ | 2017 | Wrong intervention. |
| The iTreAD project: a study protocol for a randomised controlled clinical trial of online treatment and social networking for binge drinking and depression in young people | Kay-Lambkin, FJ. Baker, AL. Geddes, J. Hunt, SA. Woodcock, KL. Teesson, M. Oldmeadow, C. Lewin, TJ. Bewick, BM. Brady, K. et al. | 2015 | Wrong intervention. |
| The long and the short of treatments for alcohol or cannabis misuse among people with severe mental disorders | Baker, A. Turner, A. Kay-Lambkin, FJ. Lewin, TJ | 2009 | Wrong patient population. |
| The MEADOW PROJECT (Mending the Effects of Alcohol and Depression on Women) and The Bridge Program (Connecting Recovery Treatment and Behavioral Health) | NCT00851669, | 2009 | Adult population. |
| The Place of Additional Individual Psychotherapy in the Treatment of Alcoholism: a Randomized Controlled Study in Nonresponders to Anticraving Medication-Results of the PREDICT Study | Berner, MM. Wahl, S. Brueck, R. Frick, K. Smolka, R. Haug, M. Hoffmann, S. Reinhard, I. Lemenager, T. Gann, H. et al. | 2014 | Adult population. |
| Therapeutic alliance and change in psychiatric symptoms in adolescents and young adults receiving drug treatment. | Rogers, N. Lubman, D. I. Allen, N. B. | 2008 | Wrong study design. |
| Therapeutic alliance predicts mood but not alcohol outcome in a comorbid treatment setting | Richardson, D. Adamson, S. Deering, D | 2018 | Adult population. |
| Therapeutic Alliance, Client Need for Approval, and Perfectionism as Differential Moderators of Response to eHealth and Traditionally Delivered Treatments for Comorbid Depression and Substance Use Problems | Kay-Lambkin, FJ. Baker, AL. Palazzi, K. Lewin, TJ. Kelly, BJ | 2017 | Adult population. |
| Therapist empathy, combined behavioral intervention, and alcohol outcomes in the COMBINE research project | Moyers, TB. Houck, J. Rice, SL. Longabaugh, R. Miller, WR | 2016 | Wrong outcomes. |
| Therapy for Undergraduate College Students Who Binge Drink and Are Depressed | NCT01632319, | 2012 | Study protocol. |
| Trajectories of adolescent alcohol use after brief treatment in an Emergency Department | Becker, SJ. Spirito, A. Hernandez, L. Barnett, NP. Eaton, CA. Lewander, W. Rohsenow, DJ. Monti, PM | 2012 | Wrong outcomes. |
| Treatment Development and Feasibility Study of Family-Focused Treatment for Adolescents with Bipolar Disorder and Comorbid Substance Use Disorders | Goldstein, Benjamin I. Goldstein, Tina R. Collinger, Katelyn A. Axelson, David A. Bukstein, Oscar G. Birmaher, Boris. Miklowitz, David J. | 2014 | Wrong patient population. |
| Treatment for Teens With Alcohol Abuse and Depression | NCT02227589, | 2014 | Study protocol. |
| Treatment of co-occurring substance abuse and suicidality among adolescents: a randomized trial | Esposito-Smythers, C. Spirito, A. Kahler, CW. Hunt, J. Monti, P | 2011 | Wrong patient population. |
| Treatment of comorbid alcohol use disorders and depression with cognitive-behavioural therapy and motivational interviewing: a meta-analysis | Riper, Heleen. Andersson, Gerhard. Hunter, Sarah B. de Wit, Jessica. Berking, Matthias. Cuijpers, Pim | 2014 | Not primary research |
| Treatment of comorbid depression and alcohol use disorders in an inpatient setting: comparison of acceptance and commitment therapy versus treatment as usual | Petersen, CL | 2008 | Adult population. |
| Treatment of Comorbid Depression and Substance Abuse in Young People | ACTRN12605000675628, | 2005 | Wrong intervention. |
| Treatment of mindfulness-based psychotherapy in adolescent inpatients with substance use disorders | DRKS00014041, | 2018 | Wrong patient population. |
| Treatment response of bipolar and unipolar alcoholics to an inpatient dual diagnosis program | Farren, Conor Kevin. Mc Elroy, Sharon | 2008 | Adult population. |
| Ultra-brief mindfulness training reduces alcohol consumption in at-risk drinkers: a randomized double-blind active-controlled experiment | Kamboj, SK. Irez, D. Serfaty, S. Thomas, E. Das, RK. Freeman, TP | 2017 | Adult population. |
| Vorvida: study protocol of a randomized controlled trial testing the effectiveness of Internet-based self-help program for the reduction of alcohol consumption for adults | Zill, Joerdis M. Meyer, Bjoern. Topp, Janine. Daubmann, Anne. Haerter, Martin. Dirmaier, Joerg | 2016 | Adult population. |
| Web-based alcohol screening and brief intervention for university students: a randomized trial | Kypri, K. Vater, T. Bowe, SJ. Saunders, JB. Cunningham, JA. Horton, NJ. McCambridge, J | 2014 | Wrong outcomes. |
| Web-Based Intervention to Reduce Substance Abuse and Depression: a Three Arm Randomized Trial in Mexico | Tiburcio, M. Lara, MA. Mart√≠nez, N. Fern√°ndez, M. Aguilar, A | 2018 | Adult population. |
| When does change begin following screening and brief intervention among depressed problem drinkers? | Baker, AL. Kay-Lambkin, FJ. Gilligan, C. Kavanagh, DJ. Baker, F. Lewin, TJ | 2013 | Adult population. |
| Who needs more than standard care? Treatment moderators in a randomized clinical trial comparing addiction treatment alone to addiction treatment plus anxiety disorder treatment for comorbid anxiety and substance use disorders | Wolitzky-Taylor, Kate. Niles, Andrea N. Ries, Richard. Krull, Jennifer L. Rawson, Richard. Roy-Byrne, Peter. Craske, Michelle | 2018 | Adult population. |
| Yoga as an adjunct treatment for alcohol dependence: a pilot study | Hallgren, M. Romberg, K. Bakshi, AS. Andr√©asson, S | 2014 | Wrong intervention. |
| Young people with co-existing mental health and drug and alcohol problems. | Hides, Leanne. Lubman, Dan I. Kay-Lambkin, Frances J. Baker, Amanda | 2007 | Preliminary results: complete results presented elsewhere. |

1. [BMJ](http://www.bmj.com/) Page MJ, McKenzie JE, Bossuyt PM, Boutron I, Hoffmann TC, Mulrow CD, et al. The PRISMA 2020 statement: an updated guideline for reporting systematic reviews. [BMJ 2021;372: n71. doi: 10.1136/bmj. n71](http://dx.doi.org/10.1136/bmj.n71) [↑](#footnote-ref-1)
